# Can high-dose tranexamic acid have a role during transurethral resection of the prostate in large prostates? A randomised controlled trial

**DOI:** 10.1080/2090598X.2021.1932125

**Published:** 2021-06-03

**Authors:** Mohamed Samir, Ahmed M. Saafan, Rania M. Afifi, Ahmed Tawfick

**Affiliations:** Department of Urology, Ain Shams University, Cairo, Egypt

**Keywords:** Benign prostatic hyperplasia, haemorrhage, high-dose tranexamic acid, transurethral resection of prostate

## Abstract

**Objectives:**

To assess the efficacy and safety of high-dose tranexamic acid (TXA) during bipolar transurethral resection of the prostate (B-TURP) in patients with large prostates compared to placebo.

**Patients and methods:**

From February 2018 to May 2020, 204 patients with enlarged prostates of 80–130 g and in need of surgical intervention were randomised into two groups. Patients in Group A underwent B-TURP and received TXA as an intravenous loading dose of 50 mg/kg over 20 min before induction of anaesthesia followed by a maintenance infusion of 5 mg/kg/h until resection was completed. The patients in Group B (placebo) received a saline infusion of a similar volume.

**Results:**

There was highly significant drop in haemoglobin in the placebo group at 4- and 24-h postoperatively compared with the TXA group (*P* < 0.001). However, there was no significant difference in the blood transfusion rate between the two groups with five patients (5.5%) in the placebo group and four (4.2%) in the TXA group requiring a transfusion (*P* = 0.74). The procedural time was significantly less in the TXA group vs the control group, at a mean (SD) of 79.93 (22.18) vs 90.91 (21.4) min (*P* = 0.001). Also, the intraoperative irrigation fluid volume and postoperative irrigation duration were significantly less in the TXA group vs the control group, at a mean (SD) of 19.21 (3.13) vs 23.05 (3.8) L and 14.75 (5.15) vs 18.33 (5.96) h, respectively (*P* = 0.001). Catheterisation and hospital stay durations were comparable between both groups (*P* = 0.384 and *P* = 0.388, respectively). No complications were recorded with use of high-dose TXA.

**Conclusion:**

High-dose TXA was effective in controlling blood loss during B-TURP in patients with large prostates, with no adverse drug reactions.

## Introduction

BPH is a pathological condition that can lead to LUTS, which affect 50% and 90% of men aged 60 and ≥70 years, respectively [1].

Different treatment options are available for BPH including watchful waiting, pharmacotherapy and surgical intervention. TURP is the surgical ‘gold standard’ treatment for BPH [2].

The prostate has a rich blood supply and because of the hyperplasia haemorrhage is the most common TURP complication [3]. During TURP about 2–5 mL/min and 20–50 mL/g of blood will be lost [4]. Haemorrhage can result from difficult intraoperative surgeon vision, prolonged operation time, a decrease in the quality of operation, the need for blood transfusion, and subsequently an increase in complications [5].

Blood loss after TURP may be due to an increase in urinary fibrinolytic activity, which facilitates the lysis of clots. This rise is caused by urokinase release by the prostate, and also the urine and urothelium contain high concentrations of plasminogen activators that stimulate the fibrinolytic system [4,6,7]. Therefore, administration of anti-fibrinolytic agents may be effective in reducing blood loss during TURP.

Tranexamic acid (TXA) is a synthetic derivative of the amino acid lysine with anti-fibrinolytic effects; it binds with both plasminogen and plasmin at lysine binding sites. This blocks the interaction of plasminogen and plasmin on the surface of fibrin and prevents the proteolytic effect on fibrin, thereby preventing the breakdown of fibrin and stabilising the blood clots, so reducing blood loss [8,9].

There is growing evidence that TXA is effective in reducing bleeding and transfusion rates in the cardiac, orthopaedic, gynaecological, transplant surgeries, and urological fields [10]. Others reported that the effect of TXA is dose-dependent and that the use of high-dose TXA is safe and effective in decreasing haemorrhage in such fields [11–15].

However, to date, using high-dose TXA in the field of urology has not been studied well and to our knowledge there is no urological research on the use of high-dose TXA in TURP. So, we conducted the present study to evaluate the safety and effectiveness of administrating intraoperative intravenous high-dose TXA for patients undergoing TURP with large prostates. The primary endpoint was to evaluate the drop in haemoglobin (Hb) and the need for blood transfusion, while the secondary endpoint was the adverse effects related to TXA use.

## Patients and methods

The study was approved by the Ethics Committee of Ain Shams University. All patients gave a written informed consent to be included in this study after an explanation was provided to them about the study’s procedures and the follow-up course. All procedures involving humans were conducted in accordance with the ethical rules of our institution’s research committee and in line with the 1964 Helsinki declaration.

The sample size was calculated using the STATA program, setting the type-1 error (α) at 0.05 and power at 95%. Results from a previous study showed that the mean (SD) postoperative Hb in the treatment group (TXA) was 11.5 (1.3) vs 10.6 (1.5) g/dL in the control group. Based on this, a sample size of 65 cases/group (130 in total) would be needed, which was raised to 80/group (160 in total) after taking in to account drop out and failure rates [15].

This randomised prospective study was carried out at tertiary care hospitals from February 2018 to May 2020. The inclusion criteria were as follows: patients aged 50–80 years with prostate sizes of 80–130 g for bipolar TURP (B-TURP). Patients known to have renal insufficiency, hepatic insufficiency, cardiac problems, neurogenic bladder, prostate cancer, urethral stricture, bladder stone, taking 5α-reductase inhibitors or whom had had previous prostate surgery were excluded. Also, patients with concomitant blood disease or coagulopathy, history of thromboembolism, epilepsy or neurological disease were excluded.

**Figure 1.**
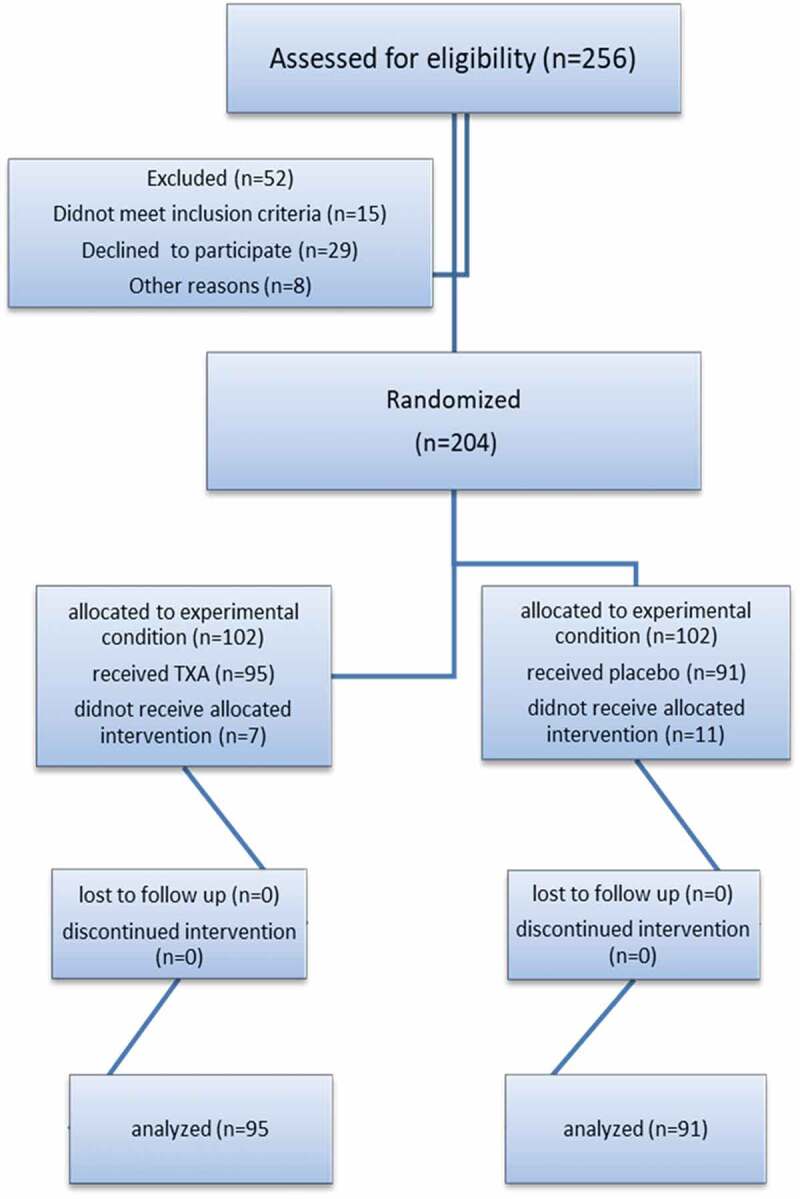
Consolidated Standards of Reporting Trials (CONSORT) flowchart.

A total of 256 patients with LUTS due to BPH were assessed for eligibility by the first author to be included in the study after taking a full history, physical examination, transrectal ultrasound, PSA measurement, urine analysis and preoperative laboratory measurements. In all, 52 patients were excluded for different reasons, 15 patients for not meeting the inclusion criteria and 29 for refusing to participate, while the remaining eight did not enter the study for other reasons (Figure 1). The remaining 204 patients were randomly divided into two equal groups of 102 patients using the closed envelope method. Group A underwent B-TURP and received high-dose TXA as an intravenous loading dose of 50 mg/kg immediately over 20 min before the induction of anaesthesia followed by a maintenance dose of 5 mg/kg/h infusion until resection was completed; and Group B underwent B-TURP and received an equal dose of saline infusion as placebo [12]. The study was simple double blinded; the same surgical team who were unaware of the difference between both groups performed all the operations.

### Operative procedure

The TXA was administered as an intravenous loading dose of 50 mg/kg immediately over 20 min before induction of anaesthesia followed by a maintenance dose of 5 mg/kg/h infusion until the TURP was completed. Each ampoule of Karpron® was manufactured by the Amoun Pharmaceutical Company (Cairo, Egypt) and contains 5 mL TXA at a concentration of 100 mg/mL. Prophylactic antibiotics were administrated at the time of anaesthesia. Under spinal anaesthesia and in lithotomy position, diagnostic cystoscopy was done to assess the urethra, size of the prostate and bladder for any abnormality and to check for the position of the ureteric orifices.

The procedure was carried out using a 26-F resectoscope (Karl Storz®; Karl Storz SE & Co. KG, Tuttlingen, Germany) with continuous flow and a U-shaped cutting loop; 0.9% normal saline was used as an irrigant. Once the device was connected, the generator (Karl Storz UH 400® surgical generator) was adjusted to 120 W for coagulation and 200 W for cutting. We started at the middle lobe then moved on to the lateral lobes. Finally, a 22-F three-way catheter was placed and postoperative irrigation started until the wash became clear and then the catheter was removed.

### Outcome measures

We compared the two groups with regards to age, prostate size in grammes, duration of the procedure, intraoperative irrigation fluid volume, resected adenoma weight, postoperative bladder irrigation period, indwelling catheter time, postoperative hospital length of stay, decrease in Hb, and blood transfusion rate. Operative time was calculated from the start of resection until the urethral catheter was inserted. The Hb level was assessed preoperatively, and at 4- and 24-h postoperatively.

We carefully monitored the patients for level of consciousness, breathing status, chest tightness, as well as urine output after surgery in order to eliminate the side-effects of the drug such as myocardial infarction, pulmonary embolism, seizures, and renal failure. Patients were examined via lower limb venous duplex ultrasound before and after surgery to exclude deep venous thrombosis (DVT).

### Statistical analysis

Continuous variables are expressed as mean (SD). Categorical variables are expressed as frequencies and percentages. The Student’s *t*-test was used to assess the statistical significance of the difference between two study groups’ means. Fisher’s exact test was used to examine the relationship between categorical variables. A statistical significance level of *P* < 0.05 was used in all tests. All statistical procedures were carried out using the Statistical Package for the Social Sciences (SPSS®), version 19 for Windows (SPSS Inc., Chicago, IL, USA).

## Results

For the preoperative data, both groups were comparable for age, prostate size and Hb as shown in Table 1.

**Table 1.** Baseline preoperative data of the patients

Variable, mean (SD)	Group		*P*
	TXA (*N* = 95)	Placebo (*N*= 91)	
Age, years	64.66 (5.87)	65.75 (5.48)	0.19
Prostate size, g	108.32 (16.64)	107.09 (16.21)	0.61
Preoperative Hb, g/dL	13.12 (1.13)	12.59 (2.37)	0.052

The decrease in the postoperative Hb was statistically highly significant in the placebo group compared to the TXA group (*P*< 0.001). However, there was no significant difference in the blood transfusion rate between the two groups, with four patients in TXA group and five in the placebo group requiring a blood transfusion, (*P*= 0.74; Table 2).

**Table 2.** The change in Hb after 4- and 24-h postoperatively and blood transfusion rate

Variable	Group		*P*
	TXA (*N*= 95)	Placebo (*N* = 91)	
Hb level, g/dL, mean (SD)			
Preoperative	13.12 (1.13)	12.59 (2.37)	0.052
4-h postoperative	10.67 (1.17)	9.09 (1.42)	0.001
24-h postoperative	10.29 (1.42)	8.91 (0.9)	0.001
Blood transfusion, *n* (%)			0.74
No	91 (95.8)	86 (94.5)	
Yes	4 (4.2)	5 (5.5)	

For the perioperative parameters, there was a statistically significant difference between the two groups in intraoperative irrigation fluid volume (mean [SD] TXA 19.21 [3.13] vs placebo 23.05 [3.8] L; *P* = 0.001), operative time (mean [SD] TXA 79.93 [22.18] vs placebo 90.91 [21.4] min; *P* = 0.001), weight of the resected prostatic adenoma (mean [SD] TXA 55.15 [12.28] vs placebo 51.47 [11.74] g; *P* = 0.038) and postoperative bladder irrigation time (mean [SD] TXA 14.75 [5.15] vs placebo 18.33 [5.96] h; *P* = 0.001). However, there was no significant difference in catheterisation time (mean [SD] TXA 51.61 [6.92] vs placebo 52.56 [7.9] h; *P* = 0.384) or hospital stay (mean [SD] TXA 55.92 [7.57] vs placebo 56.85 [7.08] h; *P* = 0.388) as shown in Table 3.

**Table 3.** Perioperative data

Variable, mean (SD)	Group		*P*
	TXA (*N* = 95)	Placebo (*N*= 91)	
Irrigation fluid, L	19.21 (3.13)	23.05 (3.8)	0.001
Operative time, min	79.93 (22.18)	90.91 (21.4)	0.001
Weight of resected adenoma, g	55.15 (12.28)	51.47 (11.74)	0.038
Postoperative irrigation time, h	14.75 (5.15)	18.33 (5.96)	0.001
Catheterisation time, h	51.61 (6.92)	52.56 (7.9)	0.384
Hospital stay, h	55.92 (7.57)	56.85 (7.08)	0.388

As regard the side-effects, no clinical symptoms or signs suggesting acute renal failure, seizures, pulmonary embolism or myocardial infarction related to the use of TXA were noticed. Also, lower limb venous duplex revealed no DVT in the TXA-treated group.

## Discussion

The prostate is a solid organ with a rich blood supply and surrounded by large venous sinuses [3]. As such, bleeding is one of the most common complications during TURP and TURP perioperative bleeding is associated with an ~4.4% rate of blood transfusion [16]. Many approaches have been used in attempts to reduce this bleeding, including catheter traction, intravenous oestrogen, intraprostatic vasopressin, oral ethamsylate, phenol solution, fibrin adhesive, preoperative 5α-reductase inhibitors, and goserelin acetate single dose [17].

When an electric current is used during TURP to remove prostatic tissue, many fibrinolytic enzymes are released into the blood circulation, thus activating the fibrinolysis system, which promotes bleeding. So, administration of anti-fibrinolytic drugs may be beneficial in reducing perioperative bleeding [6].

Multiple studies reported that TXA is effective in reducing the blood loss in different surgical fields. However, in prostate surgeries, the effect of TXA has not been established. Longo et al. [18], in a systematic review of nine studies, concluded that more studies were needed on the role of TXA in the reduction of bleeding during TURP due to limited number of studies and the high heterogeneity of the results.

Our present study revealed that blood loss was indeed decreased with the use of TXA; the 4- and 24-h postoperative results showed that Hb drop was highly significantly less in TXA group (*P* = 0.001). This is in agreement with Karkhanei et al. [2] who reported that the Hb decrease in the control group was 1.22, which was 0.93 higher than the TXA group (*P* < 0.05); and Vezhaventhan et al. [19] who reported that the total blood loss and blood loss/g resected tissue were significantly lower in patients given TXA than in the control group (*P* < 0.01). Rannikko et al. [7] reported that TXA in TURP significantly reduced the operative blood loss (128 vs 250 mL, *P* = 0.018) and also reduced the amount of blood loss/g of resected tissue (8 vs 13 mL/g, *P* = 0.020). Moreover, Longo et al. [18] reported that the blood loss was lower in the TXA group, with a standardised mean difference of −3.70 (95% CI −6.17 to −1.23; *P* < 0.001, I^2^ = 98%); however, they reported that Hb levels did not differ between the two groups after 24 h and after exclusion of a trial responsible for the absence of a difference, a small higher effect was reported favouring TXA in 24-h postoperative Hb.

In contrast, Kumsar et al. [20] reported that there was no statistically significant difference in the first day postoperative Hb loss (*P* = 0.086), which was 0.71 g/dl in the TXA group and 0.98 g/dl in the control group. However, they mentioned that the mean loss of Hb/g of resected prostate tissue was significantly lower in TXA group in comparison to the control group (1.25 vs 2.84 g; *P* < 0.001), as well as with the total Hb loss in the irrigating fluid, which was significantly lower in the TXA group (*P* = 0.018). Also, Pawar et al. [21] stated that the Hb decrease was almost the same in both groups after 24 h. However, they reported that the mean (SD) total blood loss and blood loss/g of resected tissue were significantly lower in the TXA group, at 124.6 (8.45) vs 141.05 (12.17) mL (*P* < 0.001) and 5.47 vs 5.95 mL/g (*P* < 0.05), respectively [21]. Jendoubi et al. [22] reported that intravenous TXA had no impact on perioperative blood loss in TURP. Also, Meng et al. [23,24] found that there were no significant differences in Hb concentration between the two groups and TXA had no significant impact on 24-h postoperative blood loss. However, they reported that TXA could reduce intraoperative and 4-h postoperative blood loss (*P* = 0.002 and *P* = 0.035, respectively). This may be due to the low does used in these trials.

In our present study, there was no difference in blood transfusion rate between the two groups. This was in agreement with Longo et al. [18] in TURP group where the risk ratio was 0.65 (95% CI 0.35–1.23; *P* = 0.18, I^2^ = 31%). Also, Karkhanei et al. [2], Rannikko et al. [7] and Jendoubi et al. [22] showed that there was no statistically significant difference in the blood transfusion rate.

In the present study, the difference in operative time was highly significantly shorter in the TXA group (*P* = 0.001). This can be explained by better haemostasis and improved vision with the use of TXA. This is compatible with the results of Karkhanei et al. [2], who found that there was a significant difference in operative time between the two groups, which was less in the TXA group (mean [SD] 67 [31] vs 14 [33] min; *P* < 0.05) and Rannikko et al. [7] who reported that the operating time was 36 min in the TXA group vs 48 min in the control group (*P* = 0.001). Furthermore, Vezhaventhan et al. [19], Kumsar et al. [20] and Pawar et al. [21] reported that the operative time was less in the TXA group. Conversely, Meng et al. [23] reported that there was no significant difference in the operation time between the two groups.

Rannikko et al. [7] and Pawar et al. [21] found that the volume of intraoperative irrigation fluid was significantly lower in theTXA group (*P* = 0.004 and *P* < 0.10, respectively). Also, Vezhaventhan et al. [19] reported that there was a statistically significant reduction in the amount of irrigation fluid used in the TXA group. This is compatible with our present results, as the volume of irrigation fluid was larger in the placebo group than the TXA group (*P*= 0.001). Kumsar et al. [20] reported that the total amount of irrigation fluid used was 16.34 L in the TXA group compared to 20.05 L in the control group (*P* = 0.027). While, Meng et al. [23] found no significant difference in the bladder irrigation volume of the two groups intraoperatively or postoperatively.

In our present study, the volume of resected prostatic tissue was larger in the TXA group (*P*= 0.038) and in agreement with the results of Kumsar et al. [20] and Pawar et al. [21]. Conversely, Rannikko et al. [7] reported that the amount of tissue resected between the two groups was the same (16 vs 16 g, *P* = 0.415).

We found that there was no significant difference in the catheteristion time (*P* = 0.384) and hospital stay (*P* = 0.388) between the TXA and control groups. In line with our present results, Kumsar et al. [20] reported that the duration of catheterisation and hospitalisation were the same in both groups (*P* = 0.415 and *P* = 0.218, respectively). Also, Rannikko et al. [7] and Meng et al. [23] concluded that TXA had no significant effect on catheterisation and hospitalisation times.

Although there is a theoretical concern of increased risk of thromboembolic events secondary to TXA usage, Cochrane Database concludes that TXA has no negatively affect on morbidity and mortality. ^[24]^ And there are many study generally states that use of TXA is safe and does not increase the risk of thromboembolic manifestation . ^[8, 25]^

Murkin et al and others have concluded that high dose TXA in elderly patients with cardiac problem can cause complications, like postoperative convulsions.^[26–28]^ This complication have only been demonstrated in cardiac surgery patients and may be due to blocking of GABA receptors by TXA in nerve cells that may be exacerbated by open heart surgery and a lot of study concluded that high does TXA is safe.^[12,13,14,26–30]^

The main limitation of the present study was the small sample size, the inability to assess total blood loss and mean blood loss/g of resected prostatic tissue. Further studies on the use of TXA, especially in TURP surgeries, are required.

## Conclusion

The use of high-dose TXA reduces blood loss that can lead to better surgical conditions and, consequently, shorter operative times and lower irrigating fluid volumes, without increasing the catheterisation time, hospital stay and thromboembolic complications in surgeries for large prostates.
